# Dual-functional bioactive silk sericin for osteoblast responses and osteomyelitis treatment

**DOI:** 10.1371/journal.pone.0264795

**Published:** 2022-03-02

**Authors:** Chayanee Noosak, Pavarish Jantorn, Jirut Meesane, Supayang Voravuthikunchai, Dennapa Saeloh

**Affiliations:** 1 Division of Biological Science, Faculty of Science, Prince of Songkla University, Hat Yai, Songkhla, Thailand; 2 Natural Product Research Center of Excellence, Faculty of Science, Prince of Songkla University, Hat Yai, Songkhla, Thailand; 3 Institute of Biomedical Engineering, Faculty of Medicine, Prince of Songkla University, Hat Yai, Songkhla, Thailand; 4 Center of Antimicrobial Biomaterial Innovation-Southeast Asia and Natural Product Research Center of Excellence, Faculty of Science, Prince of Songkla University, Hat Yai, Songkhla, Thailand; 5 Faculty of Medical Technology, Prince of Songkla University, Hat Yai, Songkhla, Thailand; East China Normal University School of Life Sciences, CHINA

## Abstract

Sericin, a natural protein from silk cocoon, has been reported for various biological properties in the biomaterials field. Modified forms of sericin have been studied for bone tissue engineering, while its unmodified form has been scarcely reported. Therefore, the purpose of this study was to evaluate physical and biological properties of unmodified sericin for potential use in bone surgery. Sericin was extracted from silk cocoons using a chemical-free boiling method. Sericin extract showed distinct bands with molecular weight ranging from 25 to 42 kDa including smear bands. Fourier transform infrared spectra presented characteristic peaks of amide I, II, and III, confirming the chemical composition of sericin. Based on biological activity, sericin extract at a concentration of 40 μg/mL increased the proliferation of osteoblast cells up to 135%, compared with the untreated control. Moreover, increase in antibacterial activity against *Staphylococcus aureus*, both clinical isolates and the reference strain ATCC 29213, was demonstrated for sericin extract with normal saline, while no antibacterial activity was observed for sericin with broth. It was found that sericin with normal saline showed higher zeta potential than sericin without normal saline, indicating higher system stability. This was confirmed by the average particle size of sericin extract with NaCl (3,249.3±226.1 nm) showing approximately 10 times smaller than sericin solution (29,015.9 ± 8,085.6 nm). Furthermore, sericin extract at the minimal inhibitory concentration significantly reduced the biofilm formation of *S*. *aureus* up to 95%. The study indicates biological activities of sericin, which could be applied as a dual-functional bioactive material to support bone regeneration and treat bone infections.

## Introduction

The main proteins from *Bombyx mori*’s silk cocoon threads of raw silk are fibroin and sericin. In the silk textile industry, sericin is removed from fibroin in wastewater during the processing. The global silk production statistics show that approximately 50,000 tons per year of sericin is discarded [1]. Recovery and recycling of sericin from the silk process waste could provide high scientific and economic values.

Sericin is a natural polymer composed of 18 amino acids, most of which have polar side chains such as hydroxyl, carboxyl, and amino groups, which strongly influence their physical and biological properties [2]. Sericin has interesting physical attributes of elasticity and tensile strength, and exhibits various biological activities, including antioxidant, moisturizing, antitumor, cell proliferation promoting, and collagen production supporting activities [3–7]. It has the ability to improve cell proliferation when used as a supplement in cell cultures media of human epithelial cells, human embryonic kidney transformed cells, human hepatoblastoma cells, and murine hybridoma cells [6]. In addition, sericin is presently studied for biomedical applications, especially for promoting collagen production, which is important in wound healing in the skin and accelerating osteogenesis in the bone healing process [8]. Several studies have investigated the use of sericin in wound healing applications, while bone healing has been scarcely studied. For its antibacterial activity, sericin from *Samia ricini* has been reported to affect *Escherichia coli* and *Staphylococcus aureus* [9]. Moreover, the potential of biofilm inhibition and disruption activities against *Streptococcus mutans* has been demonstrated for sericin extracted using urea and autoclaving [10].

Several methods have been performed to extract sericin, including alkaline, acidic, urea, enzymatic, and boiling. The conventional method using soap and alkaline solution is an effective method because it can completely extract sericin from the silk cocoon. However, separation of soap from sericin is a very complex process, and the application of sericin might suffer if some soap were left [11]. Acids like citric, tartaric, and succinic acid have been applied for extracting sericin. Nevertheless, both alkaline and acidic extractions can degrade sericin, damaging proteins during the extraction [12]. An extraction of sericin in urea solution with 2-mercaptoethanol has been used and proven to have lesser degrading effects on sericin. However, this method is time-consuming, and purification by dialysis is required [12]. An enzymatic extraction has been explored, but it is expensive and promotes the proteolytic hydrolysis of the primary sericin [13]. Heat extraction by boiling in water or using an autoclave is a chemical-free method to extract sericin. Important factors playing key roles in the yield of sericin are extraction time and temperature, which vary by study. Boiling sericin may cause degradation, but it still retains its properties. Among the alternative methods, boiling extraction is the preferred method for extracting sericin from silk cocoons since neither a chemical agent nor complicated equipment is required. Moreover, boiling is the easiest and least costly approach.

Recently, silk-based biomaterials become a promising candidate for biomedical application. Applying only silk or in combination with other biomaterials for developing various types of scaffolds is its practicability for use. Multiple forms of silk-based biomaterials, such as film, hydrogel, and sponge, have been developed for potential use in tissue engineering [14–16]. In bone tissue engineering, fibroin-based biomaterial has been investigated for bone repair and cartilage regeneration [17, 18], while sericin-based biomaterials, such as sericin-coated titanium and sericin nanofiber, have been reported to promote osseointegration and osteogenic differentiation [19, 20]. However, an unmodified form of sericin has been scarcely reported in this field.

This study was aimed to evaluate physical and biological properties of unmodified sericin for application in bone tissue engineering. Sericin from *B*. *mori* cocoon was extracted by boiling without using chemical agents and characterized by sodium dodecyl sulphate-polyacrylamide gel electrophoresis (SDS-PAGE) and Fourier transform infrared spectroscopy (FT-IR). In addition, the sericin extract was investigated for cytocompatibility with osteoblast cells and antibacterial/anti-biofilm activities against *S*. *aureus*, the most common cause of bone infections.

## Materials and methods

### Sericin extraction

Silk cocoons were provided by the Queen Sirikit Department of Sericulture (Narathiwat, Thailand), and they were cut into small pieces. Then, a thirty-two gram sample of the cocoon pieces was boiled in 1 L of deionized water at 100°C for 90 min. The sericin solution was filtered to remove insoluble materials, and sericin extract powder was prepared by freeze-drying using a Coolsafe Touch freeze dryer (Labogene, Bjarkesvej, Denmark).

### Sodium dodecyl sulphate-polyacrylamide gel electrophoresis (SDS-PAGE)

The molecular mass distribution of sericin extract was determined by electrophoresis under reducing conditions on 10% SDS polyacrylamide gel. To determine the molecular weights, sericin extract was mixed with loading buffer in a microtube and boiled for 10 min. Then, the sericin extract sample and protein marker PM2700 (SMoBio, Hsinchu, Taiwan) were loaded into wells. The gel was run at a voltage of 120 V for 90 min. After electrophoresis, the gel was stained with 0.25% Coomassie Brilliant Blue and de-stained in 20% methanol/10% acetic acid overnight.

### Fourier transform infrared spectroscopy (FT-IR) characterization

FT-IR spectra of commercial sericin (Sigma-Aldrich, MO, USA) and the sericin extract were analyzed using the VERTEX 70 Fourier transform infrared spectrometer (Bruker, Ettlingen, Germany). Spectra of the samples were detected in the range from 4000 to 400 cm^-1^ with a spectral resolution of 4 cm^-1^. Each spectrum was scanned 100 times, and then the average was recorded for analysis.

### Zeta potential and particle size analysis

Zeta potentials and particle sizes were measured by the ZetaPALS zeta potential analyzer (Brookhaven, NY, USA) at 25°C. The zeta potential and particle size presented were averages of 10 analyses.

### Bacterial strains

*S*. *aureus* ATCC 29213 and *S*. *aureus* clinical isolates DS18, DS27, DS84, DS87, DS88, DS92, and DS110 were provided from the Faculty of Medical Technology, Prince of Songkla University (Songkhla, Thailand). The bacteria were kept in glycerol stock and stored at -20°C. All bacterial strains were evaluated for quantitative biofilm production by a crystal violet assay according to the method described by Jantorn et al. [21].

### Determination of minimum inhibitory concentration (MIC) and minimum bactericidal concentration (MBC)

The MIC values of sericin extract against the bacteria were determined using a modified broth microdilution method according to the guidelines of the Clinical Laboratory and Standards Institute (CLSI) [22]. The overnight culture of the bacteria was cultured on tryptic soy agar (TSA) (Becton Dickinson, Le Pont de Claix Cedex, France) at 37°C for 24 h. After incubation, the bacterial colony was prepared to be in the log phase of growth at 37°C for 3–4 h in trypticase soy broth (TSB) (Becton Dickinson, Le Pont de Claix Cedex, France). The bacterial suspension was adjusted to an optical density of 0.5 McFarland standard using 0.85% sterile saline or Mueller Hinton broth (MHB) (Becton Dickinson, Le Pont de Claix Cedex, France). Then, the bacteria were diluted with MHB to obtain bacterial inoculums of 1×10^6^ CFU/mL. For sericin preparation, sericin extract powder was boiled in deionized water at 100°C, and serial 2-fold dilution was prepared using MHB. Vancomycin and sterile MHB were used as controls. The final bacterial inoculum had 5×10^5^ CFU/mL, and the final concentration of sericin extract ranged from 40–320 μg/mL. After incubation at 37°C for 16–18 h, 0.015% resazurin solution (Sigma-Aldrich, MO, USA) was added and incubated for 30–60 min to observe color change. The MIC was determined as the lowest concentration of sericin without color change. The MIC of vancomycin was interpreted following the CLSI guidelines [23]. In addition, the MBC of sericin extract was evaluated. Ten microliters of bacterial suspension from all wells with no visible growth were dropped on TSA, then incubated at 37°C for 24 h. The MBC was determined as the lowest concentration without the bacterial growth.

### Anti-biofilm activity of sericin extract

Anti-biofilm activity of sericin against *S*. *aureus* was investigated by the modified microdilution method [24]. Briefly, the overnight culture of the bacteria was grown on TSA at 37°C for 24 h. The bacterial inoculum was prepared by suspending colonies in 0.85% sterile saline to have an optical density of 0.5 McFarland standard and diluted with TSB containing 1% glucose to obtain bacterial suspension of 1×10^6^ CFU/mL. To investigate the anti-biofilm activity of sericin extract, a serial two-fold dilution of sericin extract in TSB containing 1% glucose was incubated with bacterial inoculum at 37°C for 16–18 h. The untreated bacteria were used as a control. After incubation, the culture medium was removed, and the biofilm formed in the plate was washed three times with phosphate-buffered saline (PBS) to remove non-adherent bacteria. After fixation with absolute methanol for 20 min, the plate was dried overnight at room temperature. Then, the adherent biofilm formed was stained with 2% Hucker’s crystal violet for 15 min. The excess stain was removed by washing under running tap water. Then, 33% glacial acetic acid was added to dissolve the crystal violet. Finally, the optical density (OD) was measured using a microplate reader (Multiskan Sky Thermo Scientific, Massachusetts, USA) at the wavelength of 570 nm. The percentage of biofilm inhibition was calculated using the following formula:

$$Percentage\;of\;biofilm\;formation=\frac{OD570nm\;treated}{OD570nm\;untreated}\;X\;100$$

The morphology of bacterial biofilm after treatment with sericin extract was also observed. The bacteria were cultured and prepared as mentioned above. After treatment with sericin extract at 37°C for 16–18 h, the plate was washed three times with PBS. The biofilm formed on the plate was fixed with methanol and stained with 0.1% crystal violet. Finally, the stained plate was allowed to air dry and observed using an inverted microscope (Thermo Fisher Scientific, MA, USA).

### Effect of sericin extract on osteoblast cell viability

The cytocompatibility of sericin extract to MC3T3-E1 cells was determined using the MTT assay. The MC3T3-E1 osteoblastic cell line was obtained from the Institute of Biomedical Engineering, Faculty of Medicine, Prince of Songkla University (Songkhla, Thailand). The MC3T3-E1 osteoblastic cell line was cultured in an α-modified minimal essential medium (Gibco, NY, USA) supplemented with 10% fetal bovine serum (Gibco, NY, USA), 1% antibiotic-antimycotic (Gibco, NY, USA) at 37°C in a humidified atmosphere with 5% CO_2_. Cells were grown to confluency and collected by trypsinization. Then, the cells were seeded in a 96-well plate at a density of 1×10^4^ cells/well. After 24 h of incubation, the culture medium was removed, and sericin extract (40, 80, 160, and 320 μg/mL) was added to each well and incubated at 37°C in a humidified atmosphere with 5% CO_2_ for 72 h. Untreated cells and culture media were used as controls. After 72 h of incubation, MTT solution (Thiazolyl Blue tetrazolium bromide, Thermo scientific, MA, USA) was added to each well, followed by 4 h of incubation at 37°C in a humidified atmosphere with 5% CO_2_. MTT solution was carefully discarded, and dimethyl sulfoxide was added to dissolve the formazan crystals. The absorbance readings were recorded at 570 nm using a microplate reader. Data were expressed as the percentage of viability compared with untreated cells. The percentage of cell viability was calculated using the following formula:

$$Percentage\;of\;cell\;viability=\frac{OD570nm\;treated\;cell}{OD570nm\;untreated\;cell}\;X\;100$$

### Statistical analysis

Data are presented as mean ± standard deviation. All statistical evaluations were done using SPSS version 23.0. Statistical significance was called for *p* < 0.05. Means were compared by using one-way ANOVA (analysis of variance).

## Results

### SDS-PAGE analysis

**Fig 1:**
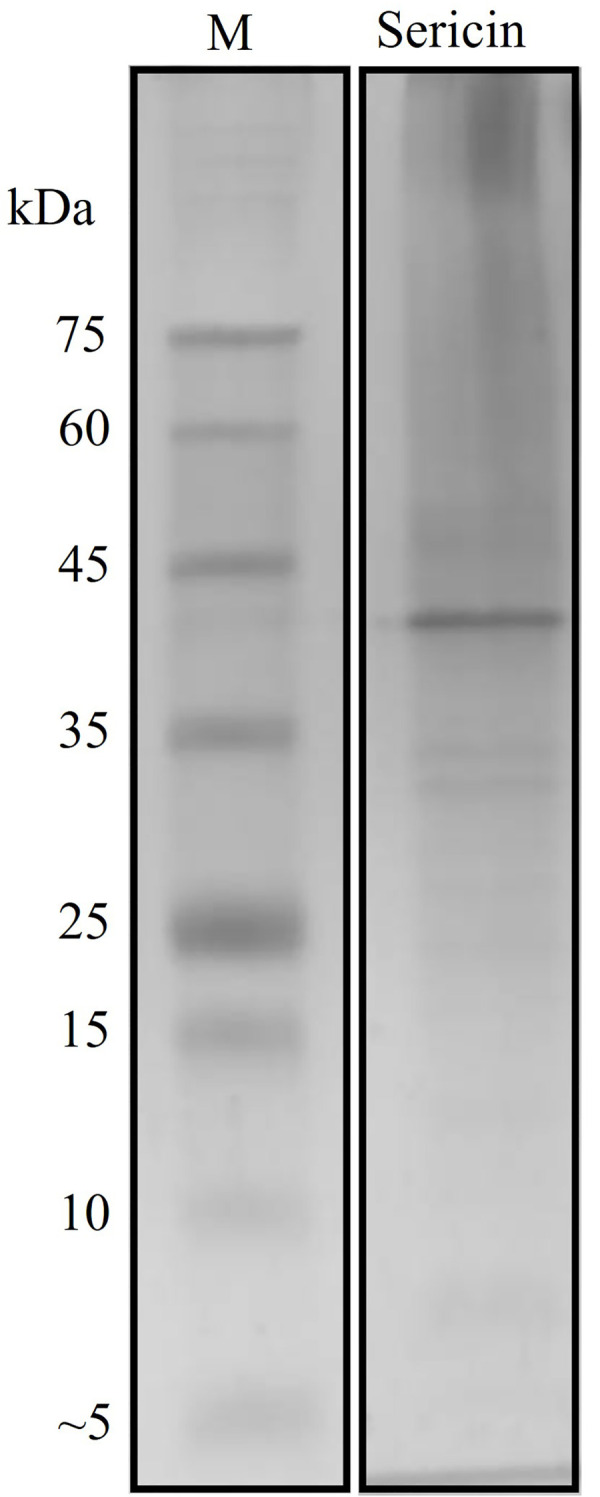
SDS-PAGE analysis of sericin extract. (M) molecular weight marker, (Sericin) Sericin extract.

The molecular weight estimation of sericin extract was performed using SDS-PAGE. The extract was separated in a 10% gel and observed by Coomassie brilliant blue staining to visualize the protein bands. Sericin extract showed distinguishable bands at 25, 33, 35, and 42 kDa, including smear bands (Fig 1).

### FT-IR spectra

**Fig 2:**
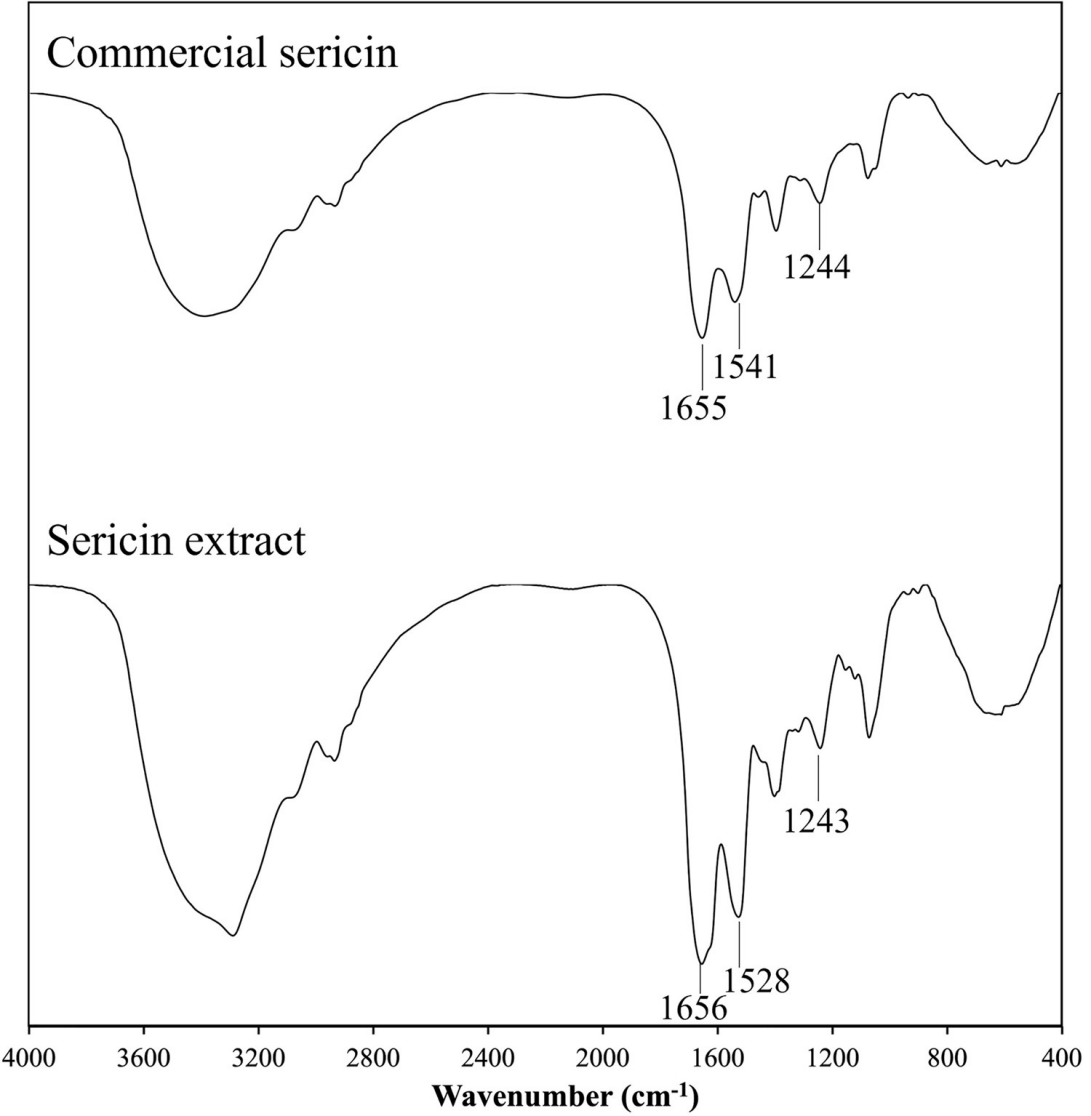
FT-IR spectra of in-house sericin extract and commercial sericin.

FT-IR spectra of sericin extract and commercial sericin were recorded using an FT-IR spectrometer. The protein molecules may present characteristic peaks from 1700 cm^-1^ to 1600 cm^-1^ corresponding to stretching of the C = O group of amide I, from 1540 cm^-1^ to 1520 cm^-1^ corresponding stretching of the N-H group of amide II, and from 1270 cm^-1^ to 1230 cm^-1^ corresponding to stretching of the C-N group of amide III [25]. As shown in Fig 2, sericin extract exhibited an amide I peak at 1656 cm^-1^, amide II peak at 1528 cm^-1^, and amide III peak at 1243 cm^-1^. FT-IR spectroscopic characteristics of the extract were very closely similar to the commercial sericin, exhibiting amide I peak at 1655 cm^-1^, amide II peak at 1541 cm^-1^, and amide III peak at 1244 cm^-1^.

### Zeta potential and particle size analysis

Sericin solution with and without normal saline was evaluated for zeta potential and particle size (Table 1). The average zeta potential of sericin with NaCl was -17.60 ± 0.68 mV, while sericin without NaCl solution posed a less negative charge of -11.91 ± 0.61 mV. Sericin with normal saline demonstrated higher zeta potential compared with sericin without normal saline, indicating higher system stability. The average particle size in sericin extract with NaCl was approximately ten times smaller than in sericin without NaCl.

**Table 1:** Zeta potential and particle size of sericin.

	Sericin with NaCl	Sericin without NaCl
**Zeta potential (mV)**	-17.60 ± 0.65	-11.91 ± 0.05
**Mean size (nm)**	3,249.3±226.1	29,015.9 ± 8,085.6

### Antibacterial activity of sericin extract

The antibacterial activity of sericin extract was evaluated by a modified broth microdilution method according to CLSI standard. Table 2 summarizes the MIC and MBC results of sericin extract against 7 *S*. *aureus* clinical isolates and reference strain ATCC 29213, with inoculums prepared using MHB and NaCl. MIC and MBC for the inoculum in broth were greater than 320 μg/mL, whereas all the bacteria prepared with sterile saline demonstrated MIC and MBC in the range of 160–320 μg/mL. Bacterial growth in the presence of vancomycin was used as antibacterial susceptibility control. The MIC and MBC for vancomycin on the inoculum prepared using MHB broth or saline ranged from 1–2 μg/ml in the quality control values (0.5–2 μg/mL) [23].

**Table 2:** Antibacterial activity of sericin extract and vancomycin against *S*. *aureus* when prepared bacterial inoculum with broth or normal saline.

		MIC/MBC (μg/mL)	
		Clinical isolates (n = 7)	ATCC 29213
Sericin	Broth	>320/>320	>320/>320
	Normal saline	160-320/160-320	160/160
Vancomycin	Broth	1-2/1-2	1/1
	Normal saline	1-2/1-2	1/1

### Anti-biofilm activity of sericin extract

**Fig 3:**
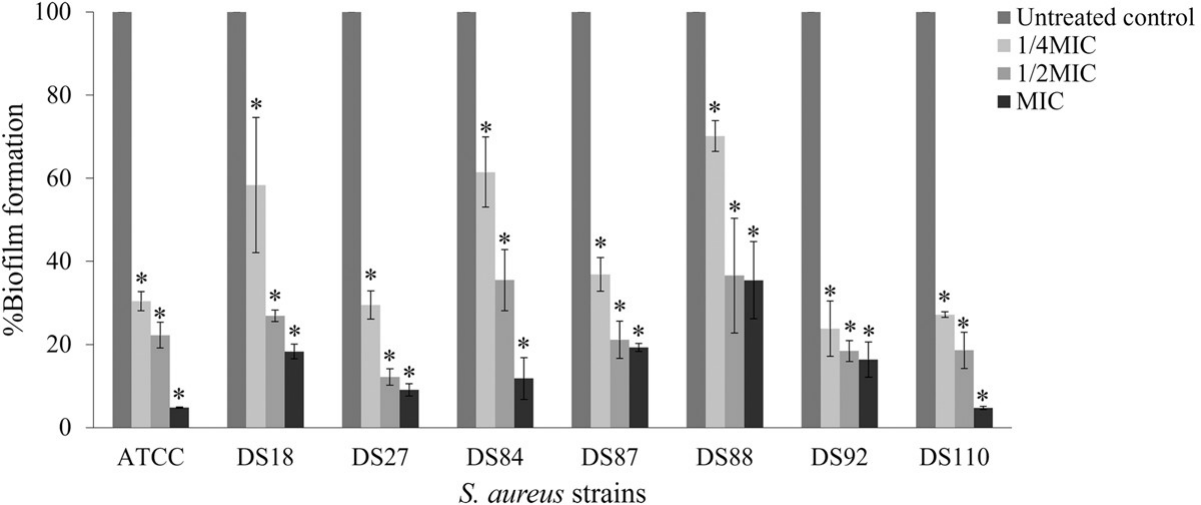
The activity of sericin extract against biofilm formation by *S*. *aureus* ATCC 29213 and clinical isolates. Statistical significance at * *p* < 0.05 compared with the untreated control.

**Fig 4:**
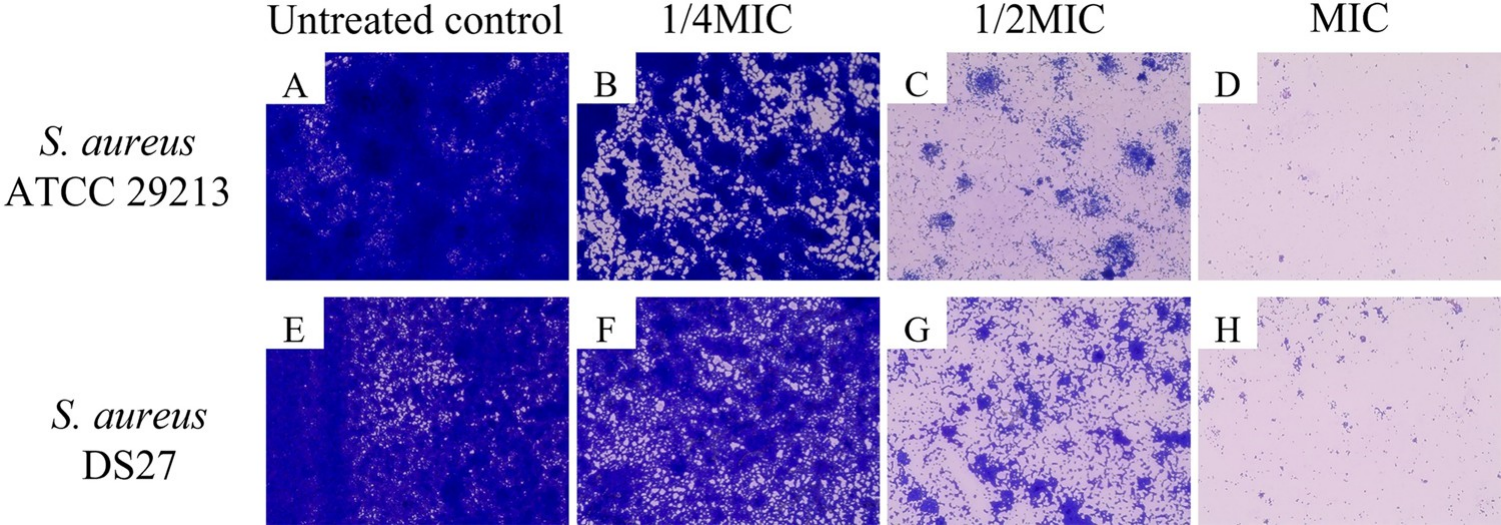
The effect of sericin extract on *S*. *aureus* ATCC 29213 and *S*. *aureus* DS27 biofilms observed using an inverted microscope at a magnification of 400X.

The effects of sericin on bacterial biofilm formation and mature biofilm were investigated. As biofilm producers, 7 *S*. *aureus* clinical isolates and reference strain ATCC 29213 were tested in this study. With concentrations at 1/4, 1/2, and MIC, sericin could inhibit biofilm formation by the bacteria in a concentration-dependent manner (Fig 3). Significant differences were observed between the treated bacteria and the untreated bacteria at all tested concentrations. Sericin extract at MIC strongly decreased biofilm formation by 65–95%. Approximately 60–90% reduction in biofilm formation was found at 1/2MIC of the extract. Biofilm formation at 1/4MIC of the extract was reduced by 30–75%. Microscopy examination was performed to confirm the inhibitory effects of sericin on biofilm formation. The biofilm was reduced after treatment with sericin compared to the untreated bacterial control shown in Fig 4. The treated biofilm appeared to have spread out, and the biofilm cluster was reduced, whereas the untreated biofilm showed a high-density cluster. The microscopy results were consistent with the quantitative analysis, which supported the inhibitory effect of sericin on biofilm formation. The sericin extract was also evaluated for its biofilm eradication activity. However, the extract had no potential to remove a mature biofilm (S1 Fig).

### Effect of sericin extract on osteoblast cell viability

**Fig 5:**
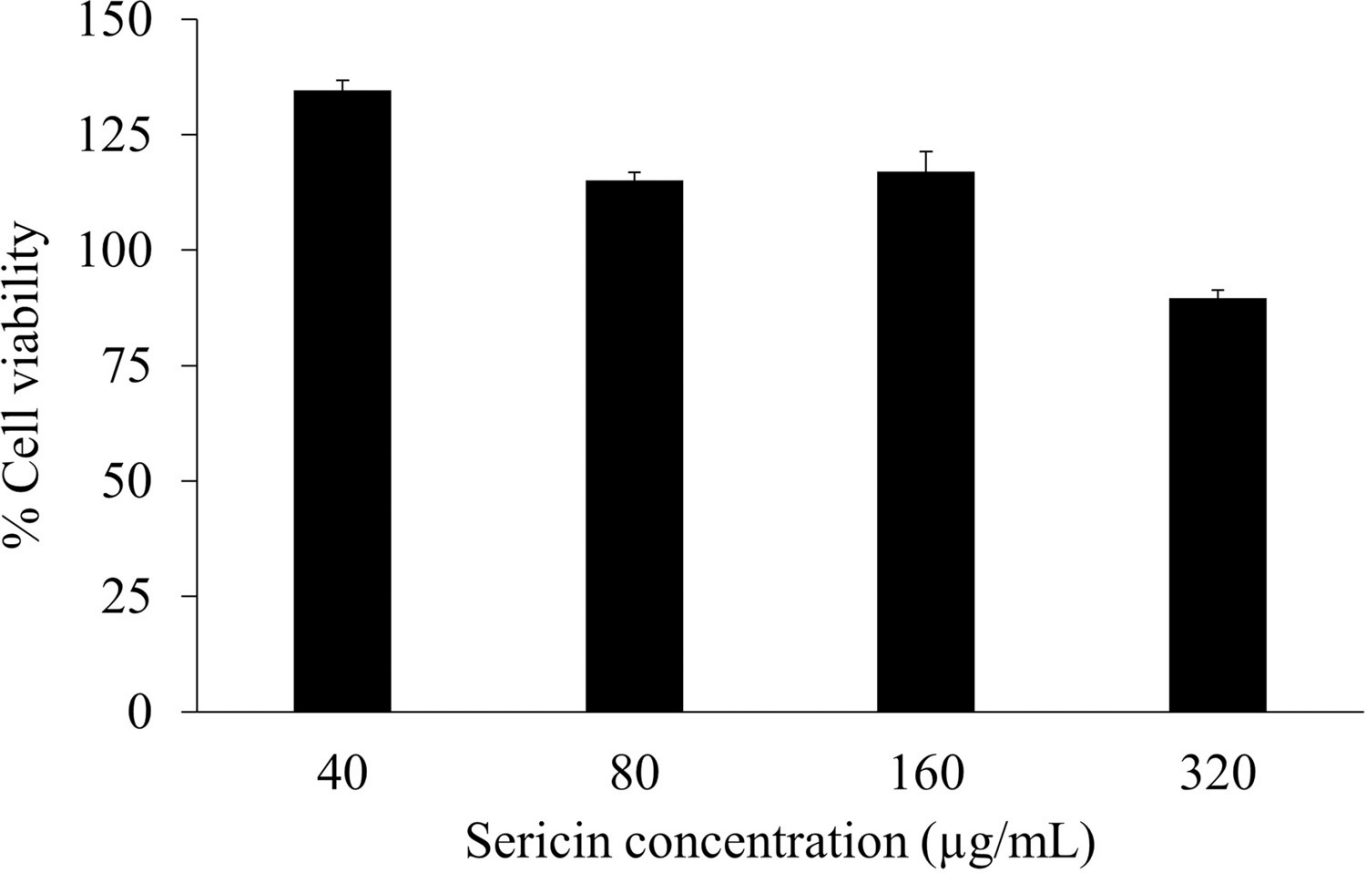
The effect of sericin extract on the viability of MC3T3-E1 cells.

Sericin extract at various concentrations was evaluated for cytocompatibility to MC3T3-E1 osteoblastic cells by an MTT assay. The cell viability of MC3T3-E1 cells after treatment with sericin extract for 72 h is displayed in Fig 5. Sericin extract at concentrations of 40, 80, 160, and 320 μg/ml showed cell viability of approximately 135%, 115%, 116%, and 90%, respectively. This obviously demonstrates that the extract had no cytotoxic effects on MC3T3-E1 cells.

## Discussion

Sericin is a waste product of the silk textile industry. It has been revalued due to its prominent properties for use in pharmacology, biomaterials, and cosmetics [26–29]. In general, sericin could be removed from silk cocoons by several methods, including chemical treatments, enzymatic methods, and heat treatments using boiling or autoclaving. However, each extraction method has several limitations. Extraction using an alkaline, acidic, or urea could chemically contaminate the product, requiring additional purification steps to obtain high-quality sericin. Enzymatic extraction is an expensive alternative and is not frequently used to extract sericin. In our study, heat extraction by boiling in water was selected to extract sericin from *B*. *mori* cocoon. Heat extraction is a safe, environmentally friendly, and cost-effective approach. In addition, it is chemical-free and does not require purification steps.

Characterization of sericin extract was performed for molecular weight distribution using SDS-PAGE and for chemical composition using FT-IR analysis. Several studies have reported that sericin molecular weight depends on the source of sericin and the extraction method. According to previous research, the molecular weight of sericin extracted by boiling in water had a broad distribution ranging from 20 to 400 kDa. In addition, the heat extraction produced a smeared band due to the degradation of polypeptide proteins into smaller molecular weight fractions [30]. The alkaline extraction showed molecular weights lower than 100 kDa [31]. The extractions by alternative methods provided products with different structures and molecular weights. In our study, the molecular weight of sericin extract was consistent with a prior study using the boiling method, which resulted molecular weights ranging from 10 to 120 kDa and presented smear protein bands [32]. However, the exact sericin molecular weights have not been reported due to the complex components of sericin affected by different extraction and processing conditions. Furthermore, FT-IR spectra were investigated to assess chemical structures in sericin extract. The FT-IR spectrum of prepared sericin extract was similar to commercial sericin. Likewise, the FT-IR results of this study were consistent with several prior studies showing amide I, amide II, and amide III in the same wavenumber ranges [2, 30, 33].

Multiple forms of sericin, including sericin-coated titanium and electrospun sericin, have shown good cytocompatibility with cell proliferation of osteoblast cells [19, 20]. This was the first time that the effects of unmodified sericin extract solution were assessed on MC3T3-E1 osteoblast cells. At the concentrations of 40, 80, and 160 μg/mL, sericin had cytocompatibility and promoted cell proliferation, showing cell viability more than 100% compared with the untreated control.

Bone infections remain a major challenge for treatments in the orthopedic field. *S*. *aureus*, the most common pathogen causing bone infections, was selected to test the antibacterial activity of sericin. According to the CLSI guidelines, the bacterial inoculum for antibacterial testing could be optionally prepared by adjusting turbidity using sterile saline or broth [22]. In this study, sericin extract exhibited antibacterial activity against *S*. *aureus* clinical isolates and a reference strain when using 0.85% normal saline to adjust the bacteria. However, sericin extract did not have antibacterial activity against the bacteria prepared with broth. Whether the bacteria were adjusted with normal saline or broth, vancomycin, a positive control, demonstrated MIC and MBC within the control range. It was supposed that sodium chloride affects antibacterial activity. Previously, sericin protein extracted with deep seawater was reported with serine as a major component [34]. This amino acid had strongly polar hydroxyl groups and may be related to the antibacterial activity of sericin. The antibacterial activity of chitosan against *Xanthomonas axonopodis* pv. *poinsettiicola* was enhanced by 0.5% NaCl. This observation was caused by the increased ionic strength of the chitosan solution [35]. Moreover, another study found that the combination of acetic acid with 3% NaCl revealed a synergistic effect against *Listeria monocytogenes*. It was hypothesized that NaCl decreased intracellular pH of *L*. *monocytogenes* [36].

We further evaluated the zeta potential and particle size of sericin solution with and without saline additive. Increase in magnitude of zeta potential of sericin with NaCl indicated stability of the solution system. The lower zeta potential implied a less stable system leading to particle aggregation. This was confirmed by larger particle sizes in sericin without NaCl. It was hypothesized that enhanced antibacterial activity of sericin might be due to a highly stable system that results from the addition of normal saline.

Biofilm is a community of bacteria encased by self-produced extracellular polysaccharide substances that help the bacteria survive in otherwise inappropriate environments and protect against host immune responses and antibiotics [37]. *S*. *aureus* commonly forms biofilms causing a major problem for clinical treatments. Sericin had significant inhibitory activity against biofilm formation by all tested *S*. *aureus* isolates, even at concentrations lower than the MIC. This result was supported by light microscopy that showed the reduction of biofilm formation. A study investigating anti-biofilm activity of sericin from different extraction methods found that the sericin extracted using urea and heat showed anti-biofilm activity against *S*. *mutans* at the concentrations of 12,500–100,000 μg/mL [10]. In our study, sericin at the concentration of 40–320 μg/mL could effectively inhibit biofilm formation by *S*. *aureus*. The study indicates sericin as a candidate anti-biofilm agent to prevent bacterial attachment on medical devices. Overall, the results emphasize that the unmodified form of sericin has dual-functional properties with cytocompatibility to osteoblast cells and antibacterial/biofilm activity against *S*. *aureus*. Our findings will lead to the development of biomaterials based on sericin for bone tissue engineering applications.

## Conclusion

In this study, sericin was extracted by boiling in water, a chemical-free and easy approach. The extract exhibited cytocompatibility with osteoblast cells. Furthermore, the sericin extract had antibacterial and anti-biofilm activities against *S*. *aureus* clinical isolates and a reference strain. This paves the way for sericin to serve as an alternative dual-functional bioactive material to promote bone cell proliferation and treat bone infection.

## Supporting information

S1 Fig The eradication effect of sericin extract on biofilm biomass of *S*. *aureus* ATCC 29213 and clinical isolates. (TIF)

S1 Raw image Gel image for Fig 1. (PDF)
