# Supplementary figures and images for: Dual-functional bioactive silk sericin for osteoblast responses and osteomyelitis treatment
Source: PLoS One. 2022 Mar 2;17(3):e0264795. doi: 10.1371/journal.pone.0264795 (PMC8890722; doi:10.1371/journal.pone.0264795)

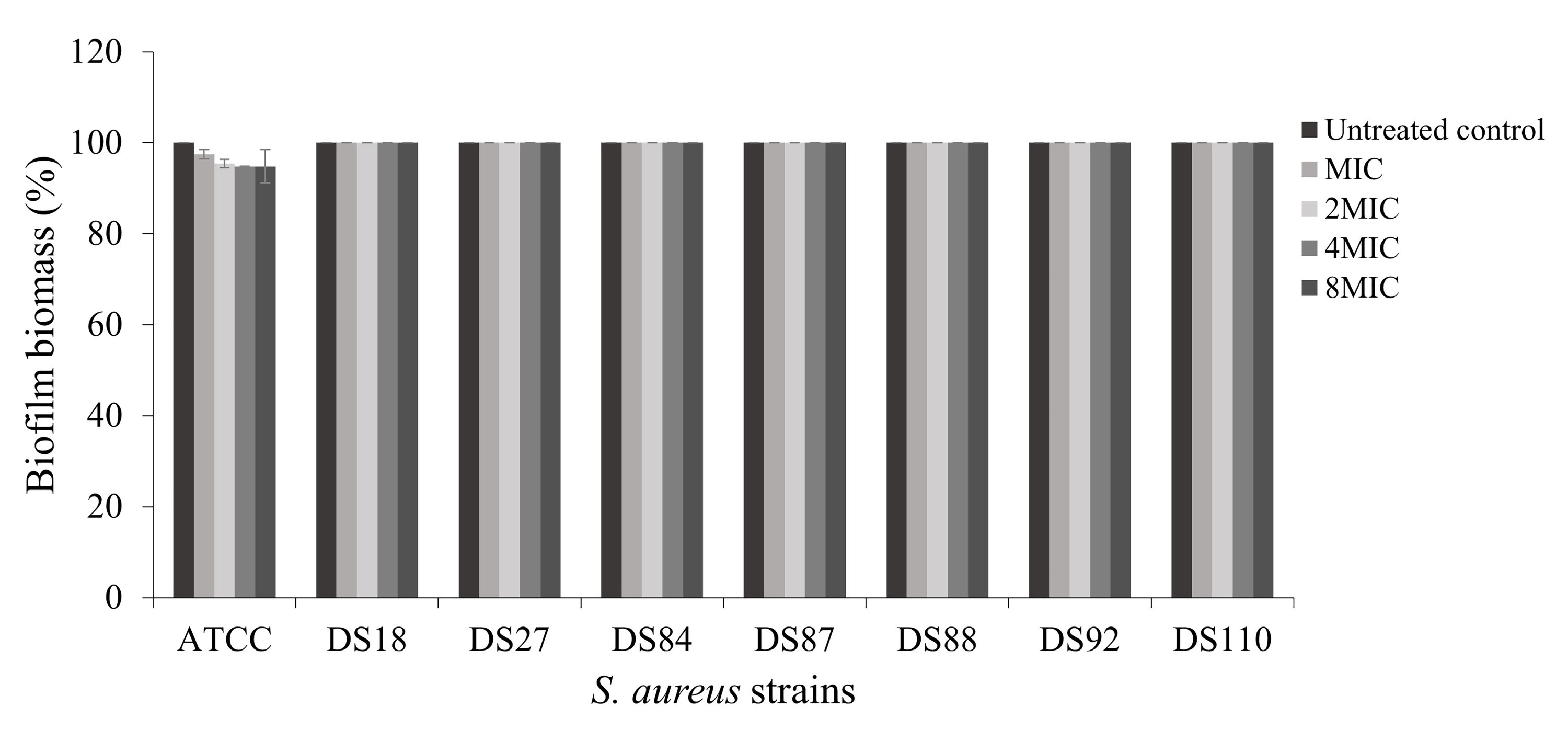

Supplement: S1 Fig — (TIF) [file pone.0264795.s001.tif]
