# Supplementary material for: Dual-functional bioactive silk sericin for osteoblast responses and osteomyelitis treatment
Source: PLoS One. 2022 Mar 2;17(3):e0264795. doi: 10.1371/journal.pone.0264795 (PMC8890722; doi:10.1371/journal.pone.0264795)
Supplement: S1 Raw image — (PDF) [file pone.0264795.s002.pdf]

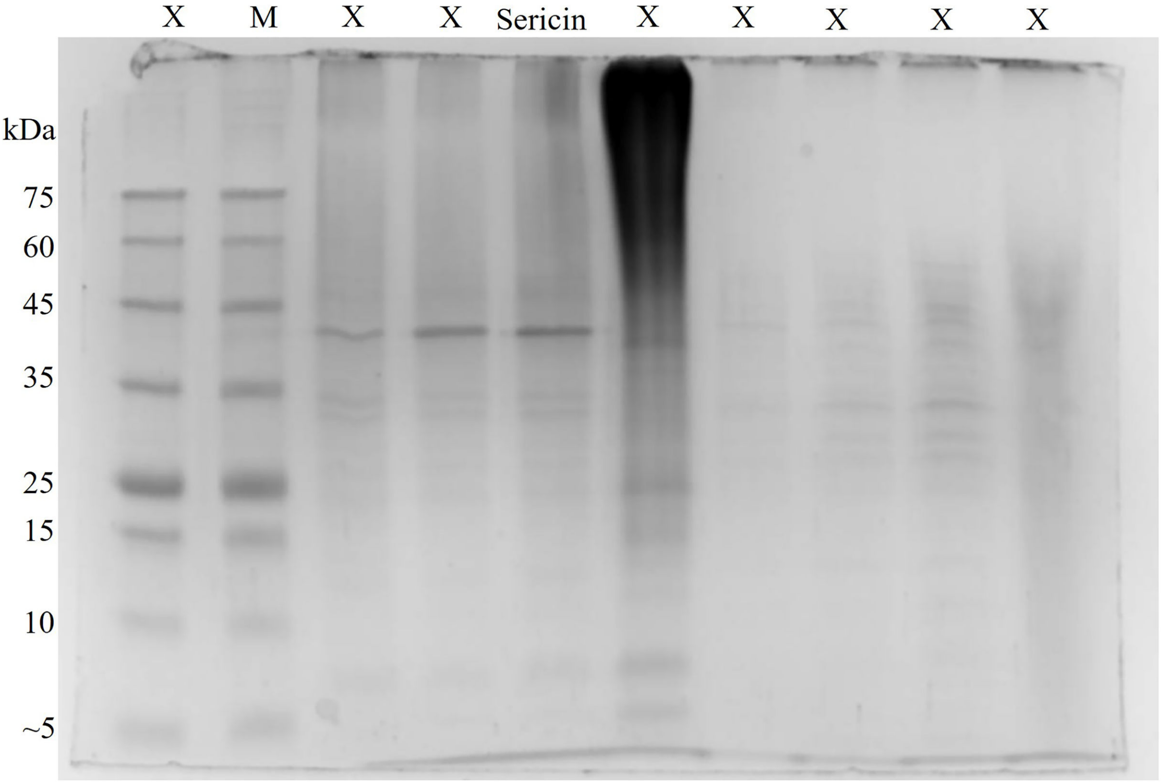

Gel image for SDS-PAGE analysis of sericin extract shown in **Fig 1**. X's indicate lanes of conditions not shown in **Fig 1**.
